# Palliative Care Evidence Review Service (PaCERS): a knowledge transfer partnership

**DOI:** 10.1186/s12961-019-0504-4

**Published:** 2019-12-16

**Authors:** Mala Mann, Amanda Woodward, Annmarie Nelson, Anthony Byrne

**Affiliations:** 1 Specialist Unit for Review Evidence, Cardiff University, Heath Park, Cardiff, CF14 4YS United Kingdom; 2 Wales Cancer Research Centre, Marie Curie Palliative Care Research Centre, Division of Population Medicine, School of Medicine, Cardiff University, Heath Park, Cardiff, United Kingdom; 3 Marie Curie Palliative Care Research Centre (MCPCRC), Division of Population Medicine, School of Medicine, Cardiff University, Heath Park, Cardiff, United Kingdom

**Keywords:** Rapid review, systematic review, evidence-based, partnership, knowledge transfer, palliative care, methodology

## Abstract

The importance of linking evidence into practice and policy is recognised as a key pillar of a prudent approach to healthcare; it is of importance to healthcare professionals and decision-makers across the world in every speciality. However, rapid access to evidence to support service redesign, or to change practice at pace, is challenging. This is particularly so in smaller specialties such as Palliative Care, where pressured multidisciplinary clinicians lack time and skill sets to locate and appraise the literature relevant to a particular area. Therefore, we have initiated the Palliative Care Evidence Review Service (PaCERS), a knowledge transfer partnership through which we have developed a clear methodology to conduct evidence reviews to support professionals and other decision-makers working in palliative care.

PaCERS methodology utilises modified systematic review methods as there is no agreed definition or an accepted methodology for conducting rapid reviews. This paper describes the stages involved based on our iterative recent experiences and engagement with stakeholders, who are the potential beneficiaries of the research. Uniquely, we emphasise the process and opportunities of engagement with the clinical workforce and policy-makers throughout the review, from developing and refining the review question at the start through to the importance of demonstrating impact. We are faced with the challenge of the trade-off between the timely transfer of evidence against the risk of impacting on rigour. To address this issue, we try to ensure transparency throughout the review process. Our methodology aligns with key principles of knowledge synthesis in defining a process that is transparent, robust and improving the efficiency and timeliness of the review.

Our reviews are clinically or policy driven and, although we use modified systematic review methods, one of the key differences between published review processes and our review process is in our relationship with the requester. This streamlining approach to synthesising evidence in a timely manner helps to inform decisions faced by clinicians and decision-makers in healthcare settings, supporting, at pace, knowledge transfer and mobilisation.

## Background

Evidence-based medicine involves the integration of the best research evidence with clinical expertise and a patient’s unique values and circumstances. It is an approach to care that encourages clinicians to use the best available evidence in combination with the individual patient’s circumstances [1]. The importance of putting evidence into practice and policy has been a focus for decades within health and social care as well as other topic areas. However, there are barriers to the uptake of evidence amongst policy-makers and clinicians, including a lack of confidence, knowledge and time [2–5]. This is particularly the case in smaller specialties such as palliative care, where pressured multidisciplinary clinicians often lack the time and/or skill sets to locate and appraise vast amounts of literature in a timeframe that aligns with clinical and service need [6, 7]. Resultant opportunity costs include inadequate underpinning of policy and operational decision-making, and lost opportunities to engage the clinical workforce with research as part of practice.

In recent years, rapid reviews have emerged as an efficient approach to synthesising evidence. They represent an alternative approach to systematic reviews that can be time-consuming, resource-intensive and costly, and may take on average from 6 months to several years [8–10]. This paper describes the approach used by the Palliative Care Evidence Review Service (PaCERS), based within the Wales Cancer Research Centre and funded by Health and Care Research Wales. The aim of the service is to provide clinicians, services and policy-makers with timely access to relevant reviews of research evidence, in support of at-pace changes to clinical practice.

Rapid reviews aim to align more closely with decision timelines and to use methodology that expedites elements of the review process. However, this must be in a manner that is scientifically robust, transparent and reproducible. Although rapid reviews are increasingly prevalent, there is no accepted standardised methodology nor an agreed definition [11–13]. In our methodology, we used the following definition, where a “[r]*apid review is defined as a review conducted within 8-10 weeks using modified systematic review methods with a highly refined research question, search carried out within limited set of databases and other sources and increasing the transparency of our methodology and explicitly summarising it for each review*” [14].

In the academic literature, various terms are used to describe rapid reviews. In an international survey of rapid review producers that took place in 2015, the terms used included ‘evidence briefs’, ‘rapid evidence synthesis’, ‘rapid systematic review’ and ‘health technology assessment’ [13]. In view of the range of terms and current practices, and in keeping with the need for transparency in adhering to key principles of knowledge synthesis [15], we set out to describe a methodology that provides a standardised and explicit framework for conducting rapid reviews of relevance to our specialty workforce. This approach delivers a detailed critique of the available literature and is presented in a stratified way for ease of access to meet stakeholder needs.

Our reviews are clinically driven and, although we are using modified systematic review methods, one of the main differences between published review processes and a PaCERS rapid review is in our relationship with the requester. We work in partnership with palliative care professionals to find the evidence they need to support changes to their clinical practice. The only reviews conducted are those requested by clinicians or decision-makers. Therefore, we carry out reviews on topics of immediate relevance to clinical practice, with the purpose to save time and resources for clinical teams and provide information in a useful format that can be implemented at pace*.* We encourage the requesters to remain involved where possible in the review process and offer training in the review methods. By only undertaking reviews in topic areas of immediate relevance to services and policy-makers, we aim to increase the potential for rapid impact on patient care and to more immediately engage the workforce with the value of research. In PaCERS, we foster a relationship between researchers and practitioners to translate and communicate evidence into practice.

## Methodology

As already indicated, PaCERS uses modified systematic review methods to produce information in a timely manner, while retaining rigour according to established systematic review methodology [16]. A Review Advisory Group (RAG) was set up to help the review team determine the constraints of a proposed review and to provide input relating to the review question and its relevance to policy and practice. The RAG consists of five members who have expertise in palliative medicine, systematic reviews and research methods as well as patient and public involvement research partners. The RAG is a fixed group that provides expertise and advice for the process of each review. Their scope is based on the criteria used for selecting rapid review projects and the 1-day workshop held to engage with stakeholders.

**Fig. 1:**
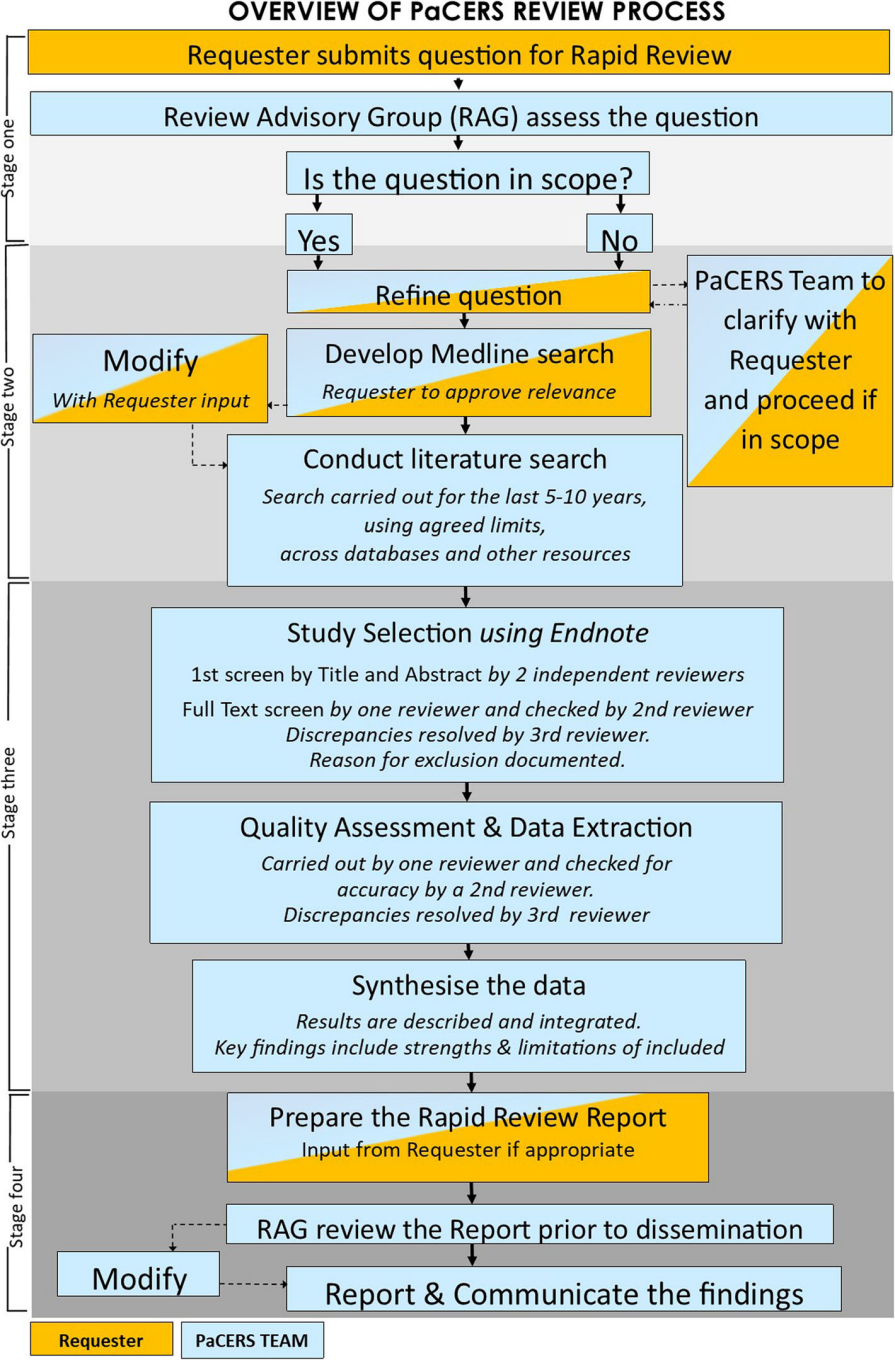
An overview of PaCERS review process

An overview of our process is outlined in Fig. 1.

A 1-day workshop was held in December 2015 for stakeholders in Wales to get a consensus on how PaCERS can best serve the palliative care community in conducting rapid reviews.

The workshop consisted of the following three sessions:

- Session 1: making a request – getting a format that makes sense
- Session 2: what would the results look like?
- Session 3: what does impact look like?

Common themes emerged (e.g. transparent reporting of the methods, tabulate results, provide key findings earlier in the report, use lay language), which gave us a better understanding of stakeholders’ interests and attitudes towards the service. This influenced key elements of our review process, in particular the structure for requesting reviews, opportunities for engagement in the review process and the structure of our reporting template. Structuring results for stakeholders is essential to facilitate the translation of findings into healthcare practice or policy.

### Stage 1: engaging with requesters and identifying the need for evidence

**Fig. 2:**
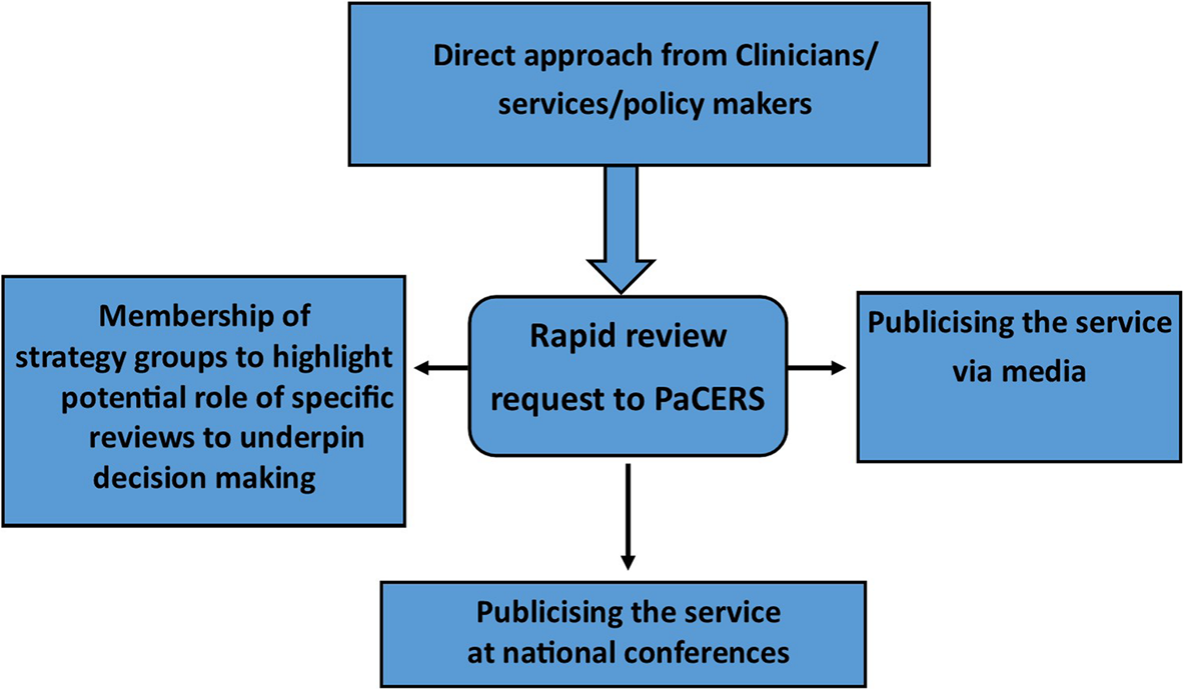
PaCERS approach to engaging clinicians and service users

The PaCERS approach to engaging clinicians and service users varies (Fig. 2). Our aim is to engage with the clinical workforce, policy-makers and across NHS organisations to undertake high quality reviews directly relevant to patient and carer needs. This enables us to close the gap between research and practice, which can be achieved through an active and engaged partnership.

Our rapid review is driven by the information captured in the rapid review request form. The request form reflects the elements found in a research protocol and acts as a guide for members of the review team. For a copy of the form, see Additional file 1. When a research question is raised, the request form is sent for completion, and this acts as the protocol to develop the rapid review. According to the PRISMA-P (Preferred Reporting Items for Systematic review and Meta-Analysis Protocols) 2015 checklist [17], it is recommended that protocols for systematic reviews or rapid reviews should be registered with PROSPERO, which is an international database of prospectively registered systematic reviews in health and social care [18]. To date, we have only registered our protocols where the review has been part of an MSc thesis with the aim to publish the review in an academic journal. However, going forward, our intention is to register all our review protocols.

Upon receipt of the request form, the first task is to check that the question is in scope with important clinical palliative care priorities, for example, the End of Life Care Delivery Plan for Wales [19], associated strategic health priorities and/or research priority exercises such as the JLA Palliative and End of Life Care Priority Setting Partnership [20]. Where more than one concurrent request is made, the RAG will prioritise based on these criteria (Table 1).

**Table 1:** Criteria used for selecting rapid review projects

• Research questions or evidence uncertainties identified and prioritised by health and social care professionals in Wales	
• Research question directly relevant to patient and carer needs and readily translated into improvements in care	
• Alignment with either End of Life Care Delivery Plan for Wales or JLA Palliative and End of Life Care Priority Setting Partnership	
• Justification by requesters that topic areas will bring noticeable benefits and evidence-based treatments to a sizable patient population	

Once the request form has been reviewed by RAG and is accepted, the next stage is to engage the requester in detailed discussion of the question. Questions posed are often very broad and lack description. Therefore, developing the question in partnership with the requester is crucial in defining the search strategy and improving the efficiency and timeliness of the review. We have found that the discourse between the review team and the requester at this early stage is important, not only in improving efficiency, but in providing teaching and training opportunities to clinical staff in review methods and developing a wider conversation within clinical teams about research engagement.

### Stage 2: defining the review process

As the team work closely with the requester to refine the wording and scope of the original question, it helps to establish the inclusion/exclusion criteria and develop the scoping search.

This process is often iterative and involves defining the elements of the question that enable the team to develop the search strategy. For effectiveness questions, the PICO (Population, Intervention, Comparison, Outcomes) framework [21] is used; for qualitative questions, the SPICE (Setting, Perspective, Intervention/phenomena of interest, Comparison, Evaluation) framework is recommended [22].

In many question formulation sessions, we have realised that these are not rigid frameworks to adhere to as some components are not relevant to the question. However, they provide structure to the iterative process of discussing and clarifying just what the research question will and will not find. Consequently, with every review question, we try to structure it using PICO or SPICE; even using only part of a framework helps to focus and facilities the literature search. Throughout the process, our ongoing communication with the end users ensures that the review is fit-for-purpose and actionable.

Synthesising evidence in the midst of complexity is challenging. Some review questions have been difficult to define, as has been the interpretation of how the intervention relates to the outcomes. Therefore, in dealing with complex interventions, the use of logic models may assist in describing the various components of interventions and the relationships between them and outlining interactions between the intervention and the system within which it is implemented [23–26]. Although logic models are often used in designing, evaluating and planning programme management and policy, we have found that this approach can help define eligibility criteria and search terms once the question has been refined, and we intend to explore this approach in future questions involving complex interventions. To date, we have only used it for one review, see example in Additional file 2.

Once the question is developed, prior to carrying out the search, we try to identify whether there are already existing systematic reviews available to answer the research question by searching the evidence sources such as the Cochrane Database of Systematic Reviews, using the topic of the rapid review; PubMed Clinical Queries, using a systematic reviews filter; and PubMed searching with the rapid review topic and the term ‘systematic reviews’.

### Stage 3: searching for information

We have developed a set of search terms relating to palliative care and cancer with input from a subject librarian working in a cancer research library on Ovid Medline (Table 2). Therefore, the only search terms that need to be identified are the terms relating to the other component of the research question, e.g. intervention or exposure, phenomena of interest, comparison or outcome.

**Table 2:** Search strategy for Ovid Medline search strategy for palliative care and cancer

1. Palliative care/	
2. Terminal Care/	
3. Terminally ill/	
4. Hospice care/	
5. (“palliative care” or “supportive care” or “hospice care” or “end of life care”).tw.	
6. ((hospice or terminal*) adj3 (care or caring or ill*)).tw.	
7. (“last year of life” or LYOL or “end of life” or “end of their lives”).tw.	
8. (end-stage disease* or end stage disease* or end-stage ill* or end stage ill*).tw.	
9. or/1–8	
10. exp. Neoplasms/	
11. (cancer* or oncolog* or neoplasm* or carcinom* or tumo?r* or malignan* or Leukemia* or lymphoma* or leukaemia*).tw.	
12. 10 or 11	
13. 9 or 12	

The search strategy is developed and conducted initially in Ovid Medline. The first 20 references are sent to the requester to check for any relevant studies. At this stage, the search strategy often needs to be refined if relevant studies are not identified. Once the strategy is finalised, the search is executed across key databases and other sources. At least six databases and supplementary sources are selected as relevant to the topic (Table 3).

**Table 3:** Bibliographic databases

Database	Database platform
CINAHL	Ebsco
Cochrane CENTRAL	Wiley
EMBASE	Ovid
Health Management Information Centre	Ovid
The Joanna Briggs Institute Evidence-Based Practice Database	Ovid
Medline	Ovid
PsycINFO	Ovid
Scopus	Thomas Reuters
Web of Science	Clarivate Analytics
Supplementary Sources	
Google Scholar – Citation tracking via Google Scholar is carried out depending on the time available.	
Electronic Table of Content of Key Journals (search minimum of two journals for last two years in relation to appropriate subject area). Key journals to hand search are selected by the requester.	
Grey Literature – Websites relevant to the topic area are searched, e.g. National Cancer Research Institute, The National Gold Standards Framework (GSF) Centre in End of Life Care, The Social Care Institute for Excellence (SCIE), INVOLVE Evidence library, European Association for Palliative care and Google.	

We identify potential sources to search depending on the scope of the question. Additionally, we test key papers to see if they are retrieved in the database combination. Grey literature sites are identified via the RAG or by the requester themselves. Experience to date has highlighted that high-quality quantitative data is not often abundant for the types of questions posed. Therefore, searches are carried out for all types of research studies without limiting to any specific study design.

Where systematic reviews or randomised controlled trials are not available, we will examine evidence from case series (≥25 patients only), case control studies, cohort studies or qualitative studies. Furthermore, where the review question is so specific and the research is limited, we have sought to include conference abstracts and extract data from service evaluations. The data sources are explicitly described in the report and analysed for quality.

The generic time limit that we employ across our rapid review search is the last 10 years, searching for studies published in Organisation for Economic Co-operation and Development (OECD) countries [27] and in English language only. We exclude doctoral dissertations and book chapters. Checking of reference lists of included studies enables us to identify any relevant studies that may have been published prior to our specified date range.

In addition to searching bibliographic databases, we carry out supplementary searching to identify unpublished research or research reported in the grey literature. We search relevant websites and the electronic table of contents of key journals for the past 2 years and scan the reference list of included studies and systematic reviews.

An adapted version of the PRISMA flow diagram [28] is used to represent the flow of information through the different phases of the review.

### Stage 4: study selection process

The search results are imported into the reference management database Endnote. Study selection by the review team is directed by eligibility criteria documented in the request form as agreed with the requester. The title and abstract screening stage of study selection is carried out by two reviewers independently. References with the decision ‘yes’ or ‘maybe’ are then retrieved in full text to be examined for eligibility.

Secondly, scanning of the full-text studies is carried out by one reviewer and checked by a second. If excluded, reasons for exclusion are recorded. At the full text stage, a record is kept in an Excel spreadsheet as to the reason for exclusion. Any discrepancies are resolved by discussion or with involvement of a third reviewer.

### Stage 5: extracting data

Data extraction forms vary from review to review since the extraction forms are tailored to the review question and the eligibility criteria documented in the request form. We pilot and refine the form to ensure that all the relevant information is captured.

Data extraction is carried out by one reviewer for all eligible studies. All data are then checked against the original article by a second reviewer. Any discrepancies are resolved by discussion or with involvement of a third reviewer.

The data captured on the data extraction forms directly informs the study characteristics table in the published review. For an example data extraction form see Additional file 3.

### Stage 6: appraising data

During the quality appraisal stage, the evidence is reviewed for its relevance, validity and results for the specific question. We assess the internal and external validity, checking the strengths and weaknesses for each paper.

In our reviews, quality assessment of the eligible studies is carried out by one reviewer and checked by another reviewer using appropriate quality assessment checklists. Any disagreements are resolved by discussion with the third reviewer to reach a consensus. We have adopted an amended version of the GATE checklist (Graphic Appraisal Tool for Epidemiological studies) [29] for which we requested permission from Professor Rod Jackson from the Section of Epidemiology & Biostatistics at the School of Population Health, Faculty of Medical and Health Sciences, University of Auckland, New Zealand. The main components we use from the GATE checklist are the study design, internal validity, study results and external validity components. We have also been using the Specialist Unit for Review Evidence (SURE) Cardiff University critical appraisal checklists; this set of checklists can be used for quality assessment of a range of different study types [30]. If several papers are found to be related to one study, these papers are grouped and only the one study is identified for quality appraisal [31].

For copies of the quality assessment checklist see Additional files 4 and 5.

### Stage 7: summarising and communicating the evidence

In order for evidence to be useful and accessed for decision-making, it must be summarised in a user-friendly format [32–34]. The challenges faced by clinicians, nurses and other professionals engaging with evidence-based practice is reported in the literature. The most common barriers stated are the lack of resources, lack of time, research barriers and lack of knowledge [3, 5, 35–37].

At the outset, when the service was established, our aim was to communicate the answer to a research question in a coherent manner. Although rapid review report formats vary greatly (e.g. from generation of a reference list through to detailed appraisal) we wished to develop a format that was explicit and comprehensive, minimising clinician time and resource in receiving and understanding the information retrieved. Our review format was informed and approved by end users at our initial workshop as being presented in a consistent and easy to read format, consisting of (1) review methods and context; (2) key findings split into three areas documenting reliability, consistency and relevance of evidence; (3) evidence implications in relation to both clinical and policy decisions; (4) PRISMA flow diagram, showing the flow of information; (5) tables of study summaries (we used the Scottish Intercollegiate Guidelines Network (SIGN) considered judgement checklist [38] for summarising the findings from each study, these include study objective, participants, interventions/comparators/methods, outcomes summary of the study results and appraisal summary); and (6) a list of included studies, bibliographic records and details of additional information available on request.

The reviews are added to the PaCERS rapid review repository, which sits within the Palliative and Supportive Care Research website [14]. When a new review is published, it is communicated via email to healthcare professionals and other decision-makers working in palliative care throughout Wales. In addition, findings have been presented at international and national conferences, where the feedback has been positive. See Additional file 6.

As William Pollard said, “*Information is a source of learning. But unless it is organized, processed, and available to the right people in a format for decision-making, it is a burden, not a benefit*” [39]. Therefore, we have resolved not only to produce the evidence in a user-friendly format but also for it to be easily accessible.

### Stage 8: demonstrating impact

Our project funders, the Wales Cancer Research Centre, just like any other research funders, expect the service to demonstrate impact. Within our request form, the question ‘Will you be able to identify and feedback to us on the impact the review has had?’ is presented. Therefore, from the very start, the requester is expected to provide feedback. When the review is completed, we send the requestor a feedback form to document the impact of the review in the weeks and months that follow, e.g. how the review was used to inform clinical practice/care provision. We cannot control impact, but we try to encourage ‘uptake’ of the research evidence, especially as PaCERS reviews are clinician led and clinically driven. For a copy of the Impact form see Additional file 7.

## Discussion

To date, we have carried out eight reviews (listed below), which are published on our Palliative and Supportive Care Research website. All reviews aim to facilitate knowledge transfer in areas of current relevance to clinicians, maximising the opportunity for new knowledge to be mobilised and implemented.

In addition, the reviews impact on conversations between researchers, clinicians and patients about the type of evidence needed, how to choose this and how to implement it to provide the right care. We have had interest from clinicians in palliative care eager to improve or change clinical practice as well as from End of Life Boards commissioners requesting rapid reviews to inform recommendations for service improvements in palliative care. Identification of knowledge gaps acts as a driver of reverse translation in generating new hypothesis-driven clinical studies. The rapid reviews conducted to date have addressed the following questions:

1. What are best practice service models in rural areas for the delivery of end of life and palliative care?
2. Does advance care planning alter management decisions made by healthcare professionals?
3. What processes decrease the risk of opioid toxicity following interventional procedures for uncontrolled pain in palliative care or cancer patients?
4. What outpatient models have proven efficacy for assessment and management of pelvic radiotherapy late effects?
5. What is the impact and effectiveness of the 7-day Clinical Nurse Specialist service on palliative care patients and their families?
6. What are the models and outcomes of public and patient involvement in cancer and palliative care research?
7. What are the perceptions about oxygen use in patients with pulmonary fibrosis and their carers?
8. What is the evidence base for the assessment and management of cancer cachexia in adults with incurable pancreatic cancer?

In PaCERS, we have attempted to incorporate a core principle of Prudent Healthcare in using evidence-based approaches to reduce inappropriate variation in care whilst using that process to facilitate the principle of “*achieving health and wellbeing with the public, patients and professionals as equal partners through co-production*”. There is potential for rapid impact when working in partnership through co-production with shared purpose and commitment.

As mentioned at the outset, there is no definitive methodology for conducting rapid reviews. In 2015, Cochrane, the global network and producer of high-quality systematic reviews of effectiveness, established the Cochrane Methods Rapid Reviews Group. Their scope was to inform rapid review methodology both within the Cochrane Collaboration and beyond. At the group’s first Colloquium open meeting in Seoul in October 2016, MM had the opportunity to present on the progress of PaCERS methodology. There are several healthcare organisations across the world producing rapid reviews using various methods in order to deliver evidence in a timely manner [38, 40–42]. As a result, there are many publications on various aspects of their methods [4, 10, 12, 43–47]. It is clear that the scope, methodology and timeline for conducting reviews differ.

## Conclusions

The PaCERS methodology aligns with key principles of knowledge synthesis in defining a process that is transparent and robust. By definition, the review process aims to shorten the steps of established systematic review methods, whilst ensuring rigour in avoiding bias during the stages of study selection, quality assessment and synthesis.

Key components of our methodology are the insistence on only undertaking reviews nominated by clinical services and policy-makers, and our direct involvement of the requester in review development. Use of this approach attempts to maximise the direct and current relevance of the evidence base to practice and the efficient production of actionable, fit-for-purpose reviews. It also creates opportunity for training in appraisal and review methods amongst a multi-professional workforce, and the facilitation of dialogue around the utility of research to clinical practice.

Additional strengths include our consensus approach to refinement of data extraction and evidence quality templates depending on the review question, and the degree to which the requester can be involved in the process whilst minimising the risk of bias. Within our review process, we attempt to work in partnership with the requester. This includes developing the question and checking the relevance of the initial search and study selection. In order to minimise potential for bias, one of the researchers from the review team is involved in every stage of the review.

We acknowledge that there are limitations within our process. We are faced with the challenge of the trade-off between the timely transfer of evidence against the risk of impacting on rigour [48]. To address this issue, we try to ensure transparency throughout the review process.

Our search is not as comprehensive as full systematic reviews since we only search for the last 10 years, in OECD countries and in the English language. Our quality assessment and data extraction are carried out by one reviewer and checked by another reviewer. Even though we are not carrying out quality assessment or data extraction independently, our methods are still stringent compared to other rapid review methods.

Due to the nature of some review questions, it has been necessary for those reviews to include service evaluations. As it is not possible to undertake quality assessment on evaluation papers, we have chosen to accept the papers to be included in the review, with a narrative commentary on their limitations. We are confident, however, that our reporting template, co-produced with clinical and academic colleagues, allows explicit recognition of these limitations whilst providing evidence in a format that is easily accessed, understood and actionable.

## Supplementary information

**Additional file 1.** Copy of the rapid review request form.

**Additional file 2.** Example of a logic model.

**Additional file 3.** Example data extraction form.

**Additional file 4.** Qualitative checklist.

**Additional file 5.** Quantitative checklist.

**Additional file 6.** List of conference and meetings.

**Additional file 7.** Impact form.

## Data Availability

Not applicable.

## Abbreviations

PaCERS: Palliative Care Evidence Review Service
RAG: Review Advisory Group
