# Supplementary material for: Palliative Care Evidence Review Service (PaCERS): a knowledge transfer partnership
Source: Health Res Policy Syst. 2019 Dec 16;17:100. doi: 10.1186/s12961-019-0504-4 (PMC6916007; doi:10.1186/s12961-019-0504-4)
Supplement: Supplementary file 1 — Additional file 1. Copy of the rapid review request form. [file 12961_2019_504_MOESM1_ESM.pdf]

Rapid Review Request Form

Canolfan Ymchwil Canser Cymru
Wales Cancer Research Centre

Palliative and Supportive Care Research

Requestor Contact Details

Name of Requester or Group:<br>*(if a group please nominate a lead)*	
Organisation:	
Contact address:	
Telephone number:	
E-mail address:	

Timeframe and relevance

Date of request:	
Deadline for completion:	
• How important/relevant will the review be for practice across Wales?<br>• How are the review findings going to be used and what organisations will be disseminating the findings?<br>• Will you be able to identify and feedback to us on the impact the review has had?	

Details of Review

Please give a brief summary of the background for the review:	
Please describe the objectives of the review?	
What key issues are you trying to explore?	
A brief description of your inclusion/exclusion criteria.	
Are you aware of any key papers?	

Rapid Review Request Form

Palliative and Supportive Care Research

Canolfan Ymchwil Canser Cymru
Wales Cancer Research Centre

A well-built research question should have at least three components. The following models will assist in shaping and focusing your question:

PICO for a quantitative question ***OR*** SPICE for a qualitative question

Choose whichever model is best suited to your question & complete the relevant table below; these are not rigid frameworks – there may be aspects that are not relevant to your question.

**Population/Problem**<br>Describe the population that you are interested in. What are the most important characteristics of the patient? What do you want us to focus on or exclude?	
**Intervention/Exposure**<br>What intervention are you interested in measuring the outcomes from.	
**Comparison/Control**<br>What is the main alternative to compare with the intervention?	
**Outcome**<br>What outcome measure/s are you interested in? What key issues are you trying to explore?	

OR

**Setting**<br>Of the research.<br>Where? In what context?	
**Perspective**<br>Of those who are experiencing the intervention or the situation of interest.<br>For who?	
**Intervention / Interest**<br>The intervention or the phenomenon that is being experienced.<br>What?	
**Comparison**<br>With those who do not receive the intervention or experience the phenomenon.<br>What else?	
**Evaluation**<br>What is being explored or evaluated in the study. How well?	

Having used one of the above models to shape and focus your research question, please present your question below:

**QUESTION**	

Please send completed form to PaCERSWCRC@cardiff.ac.uk
