# Supplementary material for: Palliative Care Evidence Review Service (PaCERS): a knowledge transfer partnership
Source: Health Res Policy Syst. 2019 Dec 16;17:100. doi: 10.1186/s12961-019-0504-4 (PMC6916007; doi:10.1186/s12961-019-0504-4)
Supplement: Supplementary file 2 — Additional file 2. Example of a logic model. [file 12961_2019_504_MOESM2_ESM.pdf]

Question: What is the impact and effectiveness of the 7 Day CNS services have on palliative care patients and their families?

Situation

Impact and effectiveness of the 7 Day CNS services on patients and their family caregivers and other healthcare professionals

INPUTS

Clinical Nurse Specialist(s) providing a Palliative Care clinical service at weekends and bank holidays 9-5pm

OUTPUTS

- Face-to face clinical assessments
- Telephone clinical assessment and advice
- Advice to Healthcare professionals
- Family support/info
- Proactive/reactive telephone call service
- Prescribing medication

OUTCOMES

- Provision of continuity care
- Patients achieving their preferred place of care/death
- Avoidance of unnecessary hospital admission
- Improved symptom management
- Prevention of drug side effects and toxicity
- Facilitated admissions to the hospice/Cancer centre
- Provision of family support at EOL
- Delivery of prescriptions
