# Supplementary material for: Palliative Care Evidence Review Service (PaCERS): a knowledge transfer partnership
Source: Health Res Policy Syst. 2019 Dec 16;17:100. doi: 10.1186/s12961-019-0504-4 (PMC6916007; doi:10.1186/s12961-019-0504-4)
Supplement: Supplementary file 3 — Additional file 3. Example data extraction form. [file 12961_2019_504_MOESM3_ESM.pdf]

APPENDIX 2

Canolfan Ymchwil Canser Cymru
Wales Cancer Research Centre
Palliative and Supportive Care Research Development

Data Extraction – Does advance care planning alter management decisions made by healthcare professionals?

Information included on this form should be comprehensive, as it will be used in the text of our review, 'Characteristics of included studies' table.

**General information**			
	1st reviewer	2nd reviewer	✓ if agree
Data extracted by:	MM		
Date:	01.12.2016		
Data extraction checked by:	AW		
Date:	01.12.2016		
Study Citation:	Wiese CH, Bartels UE, Ruppert DB, Graf BM, Hanekop GG., Wiese CHR. Prehospital emergency physicians' experiences with advance directives in Germany: a questionnaire-based multicenter study. Minerva Anestesiol 2011 Feb;77(2):172-9.	✓	
Study aims:	To identify EPs' knowledge about ethical and end-of-life decisions and their legal education and experiences concerning advance directives ADs.	✓	
Study Design:<br>*(Randomised trials, non-randomised trials, cohort study, case series (> 25 patients) and qualitative studies)*<br>*Systematic Review*<br>http://getitglossary.org/	Cross sectional study	✓	

APPENDIX 2

Include:	Exclude:
**Reason for exclusion:**	

**Country of Origin**	Germany	
**Population and Setting** (specify Health care professionals)	150 prehospital emergency physicians' EPs<br>from three emergency medical services (Braunschweig, Göttingen und Kaiserslautern	✓
**Intervention** (role of ACP)	Not applicable	
**Outcome** (any changes due to ACP in place)<br>Prevented hospital admission<br>Reduce hospitalisation<br>Empowered a doctor in A&E<br>Not to give antibiotics etc.	The presence of an Ads influenced the therapy decision in 72 EPs (all participants: 77%; experienced EPs: 30 (91%); inexperienced EPs: 43 (61%); P<0.05).	✓
**Results** (main findings)	Out of 150 questionnaires, 104 complet-ed questionnaires were returned (response rate 69%). The results<br>The presence of an AD influenced the therapy decision in 72 EPs (all participants: 77%; experienced EPs: 30 (91%); inexperienced EPs: 43 (61%); P<0.05).<br>N=86;82%) reported feeling insecure when dealing with legal questions about the validity of and obligation to uphold advance directives. They were also uncer-tain about whether advance directives influence their choice of medical therapy	13% EPs did not consider the written declaration of a will helpful. 38% EPs reported that following written advance directives in therapy decisions was obligatory (44% in the case of starting or stopping resuscitation); however, these EPs reported that therapy decisions were influenced only when the advanced directive's contents were verbally confirmed by the palliative care patient before an emergency medical situation.

	(for example, to attempt resuscitation). All 86 EPs reported the need for additional training in this area.<br>Ninety of the responding EPs (87%) said that the written declaration of a will in an advance directive helped them make therapeutic decisions (related to hospital transport or resuscitation during cardiac arrest, for example) in prehospital emergency situations.	
**Applicable to UK?**	Not sure as in Germany, fully qualified EPs and paramedics are always on board ambulances together. This is not the case in the UK	✓
**Ethical approval:**	The study was approved by the Local Ethics Committee	✓
**Conflicts of interest:**	The corresponding author and co-authors state that they have no relations with any company whose products are mentioned in the article, or with any company that sells a competing product. The current data are a part of an inaugural dissertation by DR. Another part of the questionnaire (pertaining to prehospital emergency physicians' experiences with the medical treatment of palliative care patients) was published in Palliative Medicine in 2009.	✓
**Funding sources:**	Not reported	✓

APPENDIX 2

**Notes** (limitations from author and reviewer)	The authors state that “study subjects who choose to respond to the survey may be different from those who do not respond, thus biasing the estimates”.<br>A self-administered questionnaire was used advantage receiving standardized answers but problem is inability to receive clarification.	
