# Supplementary material for: Palliative Care Evidence Review Service (PaCERS): a knowledge transfer partnership
Source: Health Res Policy Syst. 2019 Dec 16;17:100. doi: 10.1186/s12961-019-0504-4 (PMC6916007; doi:10.1186/s12961-019-0504-4)
Supplement: Supplementary file 4 — Additional file 4. Qualitative checklist. [file 12961_2019_504_MOESM4_ESM.pdf]

Additional file 4. Qualitative checklist

**Quality appraisal checklist – qualitative studies**			
In **blue** hints to help completing of the checklist			
**Study identification**<br>***(include full citation details)***			**Checked by 2nd reviewer**
**Study design:**			
**Evaluation criteria**		**Assessed by and Date :**	
		**Checked by and Date:**	
**Section 1: Theoretical approach**			
**1.1** Is a qualitative approach appropriate?<br><br>• Does the research question seek to understand processes or structures, or illuminate subjective experiences or meanings?<br>• Could a quantitative approach better have addressed the research question?			
**1.2** Is the study clear in what it seeks to do?			

• Is the purpose of the study discussed – aims/objectives/research question/s?<br>• Is there adequate/appropriate reference to the literature?<br>• Are underpinning values/assumptions/theory discussed?			
**Section 2: Study design**			
**2.1** How defensible/rigourous is the research design/methodology?<br><br>• Is the design appropriate to the research question?<br>• Is a rationale given for using a qualitative approach?<br>• Are there clear accounts of the rationale/justification for the sampling, data collection and data analysis techniques used?<br>• Is the selection of cases/sampling strategy theoretically justified?			
**3.1** How well was the data collection carried out?			

- Are the data collection methods clearly described? - Were the appropriate data collected to address the research question? - Was the data collection and record keeping systematic?			
**Section 4: Trustworthiness**			
**4.1** Is the role of the researcher clearly described?<br><br> - Has the relationship between the researcher and the participants been adequately considered? - Does the paper describe how the research was explained and presented to the participants?			
**4.2** Is the context clearly described?<br><br> - Are the characteristics of the participants and settings clearly defined? - Were observations made in a sufficient variety of circumstances - Was context bias considered			
**4.3** Were the methods reliable?			

• Was data collected by more than 1 method?<br>• Is there justification for triangulation, or for not triangulating?<br>• Do the methods investigate what they claim to?			
**Section 5: Analysis**			
**5.1 Is the data analysis sufficiently rigorous?**<br><br>• Is the procedure explicit – i.e. is it clear how the data was analysed to arrive at the results?<br>• How systematic is the analysis, is the procedure reliable/dependable?<br>• Is it clear how the themes and concepts were derived from the data?			
**5.2 Is the data 'rich'?**<br><br>• How well are the contexts of the data described?<br>• Has the diversity of perspective and content been explored?<br>• How well has the detail and depth been demonstrated?			

• Are responses compared and contrasted across groups/sites?			
**5.3 Is the analysis reliable?**<br><br>• Did more than 1 researcher theme and code transcripts/data?<br>• If so, how were differences resolved?<br>• Did participants feed back on the transcripts/data if possible and relevant?<br>• Were negative/discrepant results addressed or ignored?			
**Section 6: Summary**			
**6.1 Are the findings convincing?**<br><br>• Are the findings clearly presented?<br>• Are the findings internally coherent?<br>• Are extracts from the original data included?<br>• Are the data appropriately referenced?<br>• Is the reporting clear and coherent?			

**6.2 Are the findings and conclusions relevant to the aims of the study?**			
**6.3 Ethics - How clear and coherent is the reporting of ethics?**<br><br>• Have ethical issues been taken into consideration?<br>• Are they adequately discussed e.g. do they address consent and anonymity?<br>• Have the consequences of the research been considered i.e. raising expectations, changing behaviour?<br>• Was the study approved by an ethics committee?			
**Overall assessment**<br><br>• How clear are the links between data, interpretation and conclusions?<br>• Are the conclusions plausible and coherent?<br>• Have alternative explanations been explored and discounted?<br>• Does this enhance understanding of the research topic?<br>• Are the implications of the research clearly defined?			

**Include/Exclude**			

[1]**Appraisal form derived from 'The GATE frame: critical appraisal with pictures' by Jackson, R. et al., Evid Based Med. 2006 Apr;11(2):35-8.**

Adapted from NICE Methods for the development of NICE public health guidance (third edition) Published date: September 2012

https://www.nice.org.uk/process/pmg4/chapter/appendix-h-quality-appraisal-checklist-qualitative-studies#checklist-2
