# Supplementary material for: Palliative Care Evidence Review Service (PaCERS): a knowledge transfer partnership
Source: Health Res Policy Syst. 2019 Dec 16;17:100. doi: 10.1186/s12961-019-0504-4 (PMC6916007; doi:10.1186/s12961-019-0504-4)
Supplement: Supplementary file 5 — Additional file 5. Quantitative checklist. [file 12961_2019_504_MOESM5_ESM.pdf]

Palliative and Supportive Care Research Development

Canolfan Ymchwil Canser Cymru
Wales Cancer Research Centre

Additional file 5. Quantitative checklist

The GATE Tool

Checklist for intervention studies.

GATE CHECKLIST FOR INTERVENTION STUDIES [1]<br>(Including randomised trials, non-randomised trials, cohort studies & case series of treatment, prevention & screening)		
Study Publication (Vancouver reference)		
Appraised by (name, date):<br>Checked by (name, date):		
SECTION 1: Study Design – Methods & Numbers		
**Source population**. From which population(s), settings & locations were eligible Identified?		
**Eligible population.** What were the eligibility (inclusion/exclusion) criteria? What sampling frame / strategy was used to identify eligible from source (e.g. register, admission list)?		
**P**	**Participants**. How were they selected form the eligible populations (e.g. consecutive cases) & when?	
**E**	**Exposure(s).** Study intervention definition & how/ when/ by whom administered?	
**C**	**Comparison.** Control intervention definition & how/ when/ by whom administered?	
**O**	**Outcome(s).** Definition of 1 & 2 outcomes & how / when / by who assessed? Were they categorical or continuous?	
**T**	**Time.** If longitudinal, over what time were outcomes measured (dates)? If x-sectional, when were outcomes measured?	

SECTION 2: INTERNAL STUDY VALIDITY – Potential for bias			
**Evaluation criteria**		How well were the criteria addressed?	Quality<br>Criteria<br>Good = +<br>okay = ~<br>poor = X<br>nr = not reported<br>**+ ~ x nr**
Participants	**Eligible: Inclusion & exclusion criteria:** sufficient detail (could be replicated)?		
	Applied consistently (e.g. multiple cohorts or multi-centre study)?		
	Appropriate given study objectives (e.g. at relevant level of risk)?		
	**Participant selection from eligible:** sufficient detail on sampling frame/strategy?		

	Appropriate to study objectives (e.g. volunteers, representative sample)?		
	Exposure (i.e. intervention) / Comparison definitions: - sufficient detail (could be replicated)?		
	- was there a comparison group? (if not, study is a case series)		
	Did investigators determine Exp/Comp allocation (i.e. trial or experiment) or measure Exp/Comp (i.e. non-experimental cohort study)?		
	If trial, was allocation to Exp/Comp randomised?		
	Were participants blind to exposures?		
	Were study staff blind to exposures?		
	If trial & no blinding, was allocation method concealed from study staff & participants?		
	Was Exp/Comp allocation/measurement applied consistently to all participants?		
	Were Exp & Comp Groups (EG & CG) similar at baseline (e.g. if trial was randomisation successful)?		
	If groups not similar, were differences addressed in analyses/interpretation (e.g. multivariate analyses)?		
	Were there likely to be residual differences between Exp/Comp groups that could have important effects on outcomes (i.e. confounding)?		
	Was compliance/adherence to Exp/Comp measured? Re-measured during follow-up?		
	What was the % compliance/adherence?		
	Was level of compliance likely to cause important bias?		
	Did any of the CG receive the exposure (contamination)? What %?		
	Did any of the EG receive the comparison (contamination)? What %?		

	Was there sufficient contamination to cause important bias?		
	Aside from study Exp/Comp were EG & CG treated equally (co-intervention)?		
	Was there sufficient co-intervention to cause important bias?		
Outcomes & Time	Outcome definitions: - sufficient detail (could be replicated)?		
	- Objective & valid measurements?		
	- assessed blind to Exp/Comp allocation?		
	- applied consistently (eg if multi-centre study)?		
	Was follow-up long enough to detect important effects?		
Analyses	Were all participants initially allocated, accounted for at study conclusion?		
	What % lost to follow-up (after allocation)? At each stage of study?		
	Was loss to follow-up sufficient to cause important bias?		
	Were all participants analysed in groups they were initially allocated to (intention-to-treat)?		
**Summary Quality Score for validity: how well did the study minimise bias? (+ or ~ or x)**			

SECTION 3: STUDY RESULTS - magnitude & precision		
Were **Exposure Group Occurrence** (EGO) & **Control Group Occurrence** (CGO) given or possible to calculate for all 1º and 2º outcomes?		
Were all relevant effect estimates given or possible to calculate for all outcomes (e.g. RR, RD)?		
Was the precision of effect estimates given or possible to calculate for all outcomes (i.e. CLs & or p-values)? – see Note 3 at end of Checklist.		
Were the main effect estimates statistically significant?		
If effect estimates NOT statistically significant, were power calculations given or possible to calculate? – see Note 3 at end of Checklist.		
If effect estimates NOT statistically significant was power sufficient (e.g. > about 70-80%) to identify important effects?		

Canolfan Ymchwil Canser Cymru
Wales Cancer Research Centre
Palliative and Supportive Care Research Development

If study stopped early, were interim analyses and stopping rules well described?		
If multi-centre study or multivariate analyses, were results similar in each centre / all strata?		
**Summary Quality Score for study results: was interpretable data given/possible to calculate for measures of occurrence, estimates of effect & precision? (+ or ~ or x)**		

SECTION 4: EXTERNAL STUDY VALIDITY – applicability & generalisability			
Participants	Were descriptions of settings & locations of source population, eligible populations & sampling frame / strategy sufficient to determine generalisability?		
	What % of eligible participated?		
	What were the reasons for non-participation?		
	Were participants representative of eligible population?		
Exp / comp	Were study exposures (i.e. interventions) feasible and affordable in usual practice?		
	Was management in the Comparison Group similar to usual practice?		
Outcomes	Were all important outcomes considered: benefits & harms? (e.g. not just surrogate outcomes)?		
	Was it possible to determine the balance of benefits & harms of study exposures (i.e. interventions)?		
**Summary Quality Score for study applicability (+ or – or x)**<br>*Criteria quality scores: + = good, ~ = okay, x = poor, nr = not reported		**a) Could applicability be determined?**	
		**b) Are results generalizable to usual practice**	

**Include/Exclude** (if excluded please state the reason)	

Adapted from: Jackson R, Ameratunga S, Broad J, et al. The GATE frame: critical appraisal with pictures. Evidence-based medicine 2006;11(2):35-8. doi: 10.1136/ebm.11.2.35
