# Supplementary material for: Palliative Care Evidence Review Service (PaCERS): a knowledge transfer partnership
Source: Health Res Policy Syst. 2019 Dec 16;17:100. doi: 10.1186/s12961-019-0504-4 (PMC6916007; doi:10.1186/s12961-019-0504-4)
Supplement: Supplementary file 6 — Additional file 6. List of conference and meetings. [file 12961_2019_504_MOESM6_ESM.pdf]

**Additional file 6: Conference and meetings**
Palliative and Supportive Care Rapid Review Workshop Cardiff, Wales 14 December 2015.
Gee P, Woodward A, Nelson A, Mann M, Byrne A *Palliative Care Evidence Review Service (PaCERS) :A rapid systematic approach to identifying high quality evidence on palliative care.* The Annual Marie Curie Palliative Care Research Conference, 2016, London, UK.
Mann MK. *PaCERS Progress.* Cochrane Methods Rapid Reviews Group. 24th Cochrane Colloquium 23-27 October 2016, Seoul, South Korea Available at: http://methods.cochrane.org/rapidreviews/welcome. Accessed January 2018.
Mann MK. *Evidence into practice and policy through collaboration in Wales* What Works Global Summit (WWGS) 26-28 September 2016, London, UK
Sui J., Mann M, Woodward A, Gee P. Dolwani, Byrne A. *A Rapid Review: What is the most effective treatment in achieving early haemostasis and preventing or delaying re-bleeding.* Multinational Association of Supportive Care in Cancer, 22-24 June 2017, Washington DC, USA.
Mann MK., Woodward A, Nelson A, Byrne A . 2017, *Using evidence to improve lives: a rapid systematic approach* Global Evidence Summit, 13-16 September 2017, Cape Town, South Africa.
Mann MK. *PaCERS approach: Co-Production*. Wales Cancer Conference, 23 November 2017, Cardiff, Wales
Mann MK., Woodward A, Nelson A, Islam I, Byrne A. 2018*, Identifying existing models of public and patient involvement in palliative care research in cancer patients for better health decisions*, 25th Cochrane Colloquium, 16-18 September 2018, Edinburgh, Scotland, UK.
Mann MK., Nelson A, Woodward A, Islam I, Byrne A. 2018, *Evidence into practice: novel approaches to underpinning evidence-based innovations in care***.** The Annual Marie Curie Palliative Care Research Conference, 2018, London, UK.
Mann MK. *Thinking globally, acting locally: Palliative Care Evidence Review Service (PaCERS)*. The Wales Centre for Evidence Based Care Joanna Briggs Institute (JBI) European Regional Symposium, 3 May 2019, Cardiff, Wales, UK.
