# Supplementary material for: Palliative Care Evidence Review Service (PaCERS): a knowledge transfer partnership
Source: Health Res Policy Syst. 2019 Dec 16;17:100. doi: 10.1186/s12961-019-0504-4 (PMC6916007; doi:10.1186/s12961-019-0504-4)
Supplement: Supplementary file 7 — Additional file 7. Impact form. [file 12961_2019_504_MOESM7_ESM.pdf]

Palliative and Supportive Care Research Development

Canolfan Ymchwil Canser Cymru
Wales Cancer Research Centre

Palliative Care Evidence Review Service (PaCERS)

Rapid Review:

Name of Requester or Group:<br><br>*(if a group please nominate a lead)*	
Organisation:	
Contact address:	
Telephone number:	
E-mail address:	

Questions	Before Review[1]	Questions	Follow up
**How are the review findings going to be used?**		**Did the review findings help address how you were aiming to uses them?**	
**How important/relevant will the review be for practice across Wales?**		**How important has the review findings been in changing practice across Wales?**	
**Will you be able to identify and feedback to us on the impact the review has had?**		**What are the specific impacts that have materialised from the rapid reviews?**	

Please send completed form to PaCERSWCRC@cardiff.ac.uk

[1] Information captured from the Rapid Review Request form.
